# Conservative Treatment Achieving Bone Union in a Displaced Wright and Cofield Type B Periprosthetic Humeral Fracture After Reverse Shoulder Arthroplasty: A Case Report

**DOI:** 10.7759/cureus.104942

**Published:** 2026-03-09

**Authors:** Hiromitsu Tsuge, Kazuhiro Ikeda, Shotaro Teruya, Shinzo Onishi

**Affiliations:** 1 Department of Orthopedic Surgery, Institute of Medicine, University of Tsukuba, Tsukuba, JPN

**Keywords:** conservative treatment, elderly patients, periprosthetic humeral fractures, reverse shoulder arthroplasty, reverse total shoulder arthroplasty

## Abstract

Periprosthetic humeral fracture is a serious complication after reverse shoulder arthroplasty (RSA). Wright and Cofield classification type B fractures occur around the tip of the humeral stem. In these fractures, the intramedullary canal is occupied by the stem and cement, which reduces endosteal blood supply and makes fracture healing more difficult. As a result, surgical management, such as open reduction and internal fixation or revision arthroplasty, is commonly recommended when displacement is present. However, these procedures are highly invasive and may pose substantial perioperative risk in elderly patients.

An 82-year-old woman with multiple comorbidities underwent bony increased offset RSA (BIO-RSA) using a cemented humeral stem (Aequalis Ascend Flex; Stryker, Kalamazoo, USA). At postoperative year 4, she fell, which resulted in a displaced Wright and Cofield type B periprosthetic humeral fracture (type B fracture). Although valgus angulation progressed to 20° and the stem tip migrated medially, she had minimal pain and preferred nonoperative management due to her high surgical risk. Radiographs demonstrated preservation of cortical integrity on the lateral, anterior, and posterior aspects. Progressive callus formation was observed, and at one year after injury, bridging callus confirmed bone union. Forward elevation reached 100°, and she remained pain-free in her daily activities.

Despite concerns that type B fractures are at risk for impaired union due to loss of endosteal blood supply, this case achieved bone union with conservative treatment. Preserved cortical continuity on three cortices likely maintained periosteal blood flow, which may have compensated for compromised intramedullary circulation. These findings suggest that nonoperative treatment may be feasible in selected patients, particularly when cortical integrity is preserved.

Conservative treatment can achieve bone union even in displaced Wright and Cofield type B periprosthetic humeral fractures after RSA. Careful evaluation of cortical continuity on orthogonal radiographs, together with patient factors such as age and comorbidities, is essential when determining the optimal management strategy.

## Introduction

Periprosthetic humeral fracture is a serious complication after reverse shoulder arthroplasty (RSA). It can markedly impair shoulder function and reduce a patient's quality of life [1].

According to the Wright and Cofield classification, type B fractures occur around the tip of the humeral stem [2]. In these fractures, the intramedullary canal is occupied by the stem and cement. This can compromise the local blood supply and is disadvantageous for fracture healing [2]. Therefore, when the fracture site is displaced, surgical treatment such as open reduction and internal fixation or revision arthroplasty is commonly recommended [1,3-5]. However, these surgical procedures are highly invasive and may be associated with significant perioperative risks, particularly in elderly patients.

We encountered a case of an elderly woman who sustained a displaced Wright and Cofield type B periprosthetic humeral fracture (type B fracture) after RSA. Contrary to the existing literature, bone union was achieved with conservative treatment. We report this case as it provides important insights into the indication for conservative management of this fracture type.

## Case presentation

**Figure 1:**
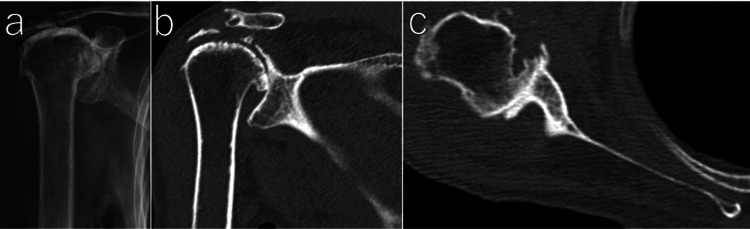
Preoperative radiological imaging Anteroposterior radiograph (a) showing cuff tear arthropathy (Hamada grade 5). Coronal oblique CT image (b) demonstrating glenoid erosion (Favard type E3). Axial CT image (c) demonstrating glenoid morphology (Walch type A2).

An 82-year-old woman presented with right shoulder pain for approximately two years. Her medical history included idiopathic thrombocytopenic purpura, chronic kidney disease, chronic heart failure, and hypertension. Her medications included hydroxyurea, amlodipine, celecoxib, teprenone, and mecobalamin. She was not receiving any osteoporosis medications. She had night pain and activity-related pain. The range of motion was restricted: forward elevation was 30°, abduction was 60°, external rotation at the side was 0°, and internal rotation behind the back reached the L5 level. Radiographic evaluation demonstrated cuff tear arthropathy with glenoid bone loss (Figure 1).

**Figure 2:**
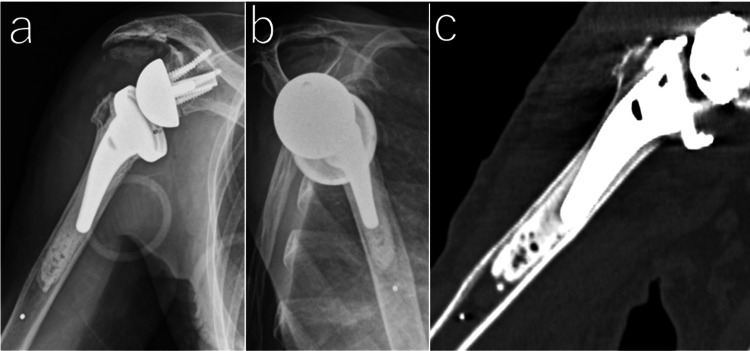
Postoperative radiological imaging Anteroposterior view (a) and scapular-Y view (b) showing adequate implant placement. Coronal CT image of the humerus (c) showing adequate cement filling.

The patient underwent bony increased offset RSA (BIO-RSA) using the deltopectoral approach. The humeral implant was an Aequalis Ascend Flex humeral stem (Stryker, Kalamazoo, USA), which was used with cement. Autologous bone graft (7 mm thickness) from the humeral head was used for the glenoid bone loss under the baseplate (Figure 2).

Postoperative course

At postoperative year 1, the range of motion improved: forward elevation was 150°, external rotation at the side was 0°, and internal rotation behind the back reached the L5 level. She did not have symptoms until postoperative year 4.

Postoperative year 4

**Figure 3:**
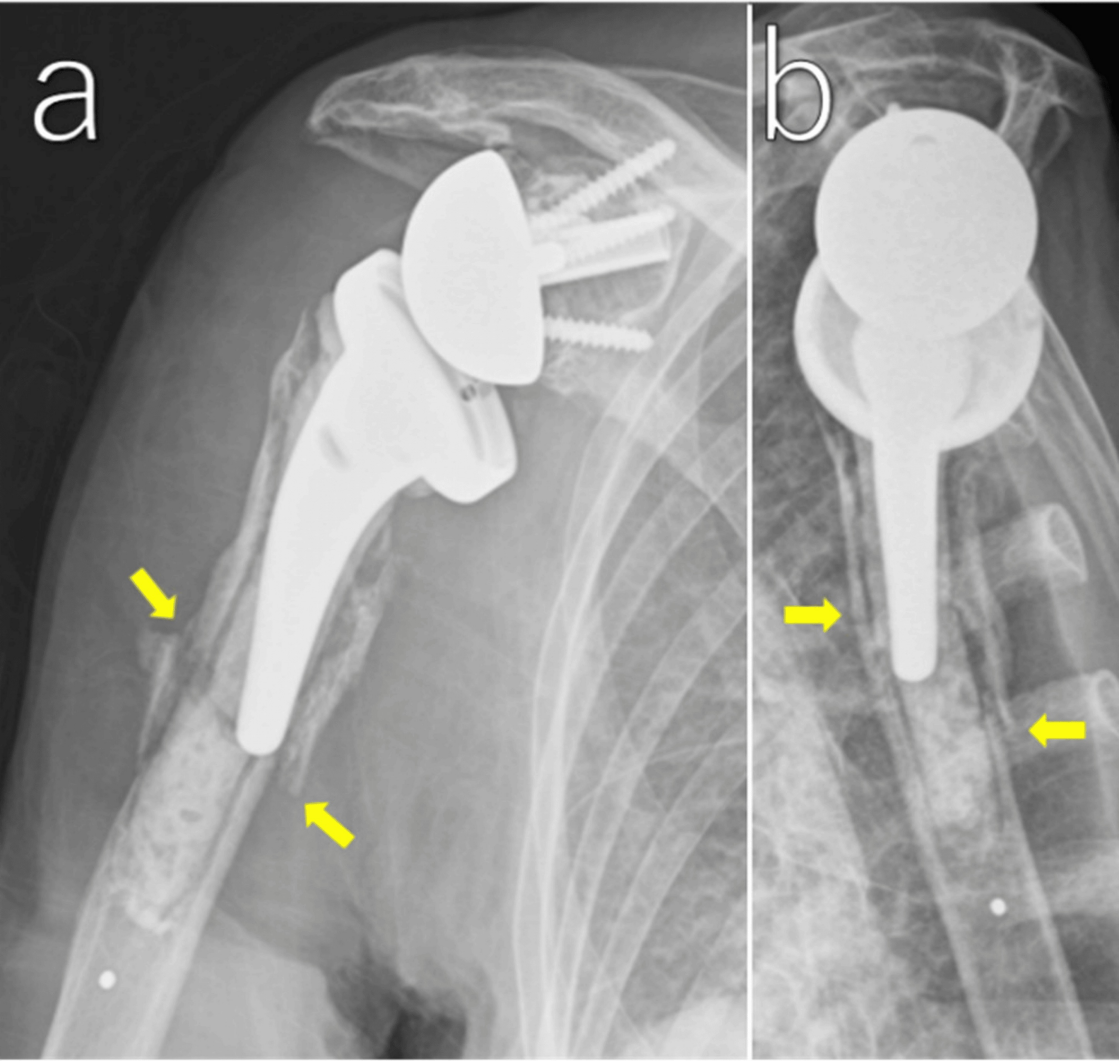
Plain radiographs at the first visit after injury Anteroposterior radiograph (a) showing a transverse periprosthetic fracture at the stem tip with minimal displacement (arrows). Scapular-Y view (b) showing minimal displacement (arrows).

The patient fell and sustained pain in her right upper arm. Plain radiographs demonstrated a Wright and Cofield type B periprosthetic humeral fracture. There was no obvious displacement and no loosening of the humeral stem. Therefore, we chose conservative treatment with sling immobilization (Figure 3).

Two weeks after injury

**Figure 4:**
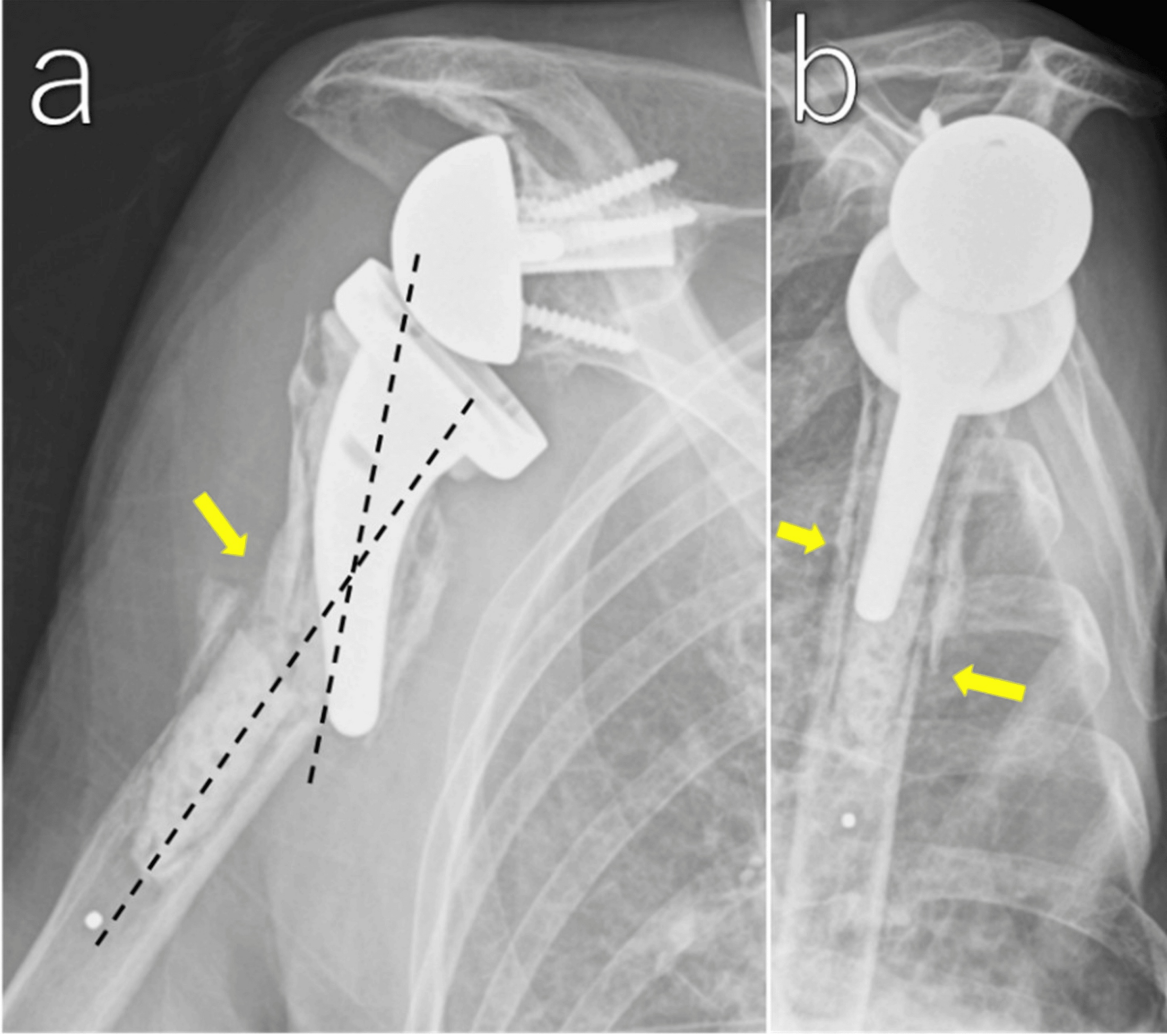
Plain radiographs at two weeks after injury Anteroposterior view (a) showing 20° valgus angulation with lateral cortical hinge preservation (arrow). Scapular-Y view (b) demonstrating maintained alignment (arrows).

Radiographs demonstrated progression of displacement with approximately 20° valgus angulation of the distal fragment. The stem tip had migrated beyond the medial cortex into the extramedullary space. On the other hand, the alignment was preserved in the scapular-Y view (Figure 4). Due to the displacement, we recommended surgical fixation. However, the patient strongly preferred conservative treatment because her pain was mild. Considering her advanced age and high perioperative risk due to comorbidities, and her preference, we continued conservative treatment.

Four weeks after injury

**Figure 5:**
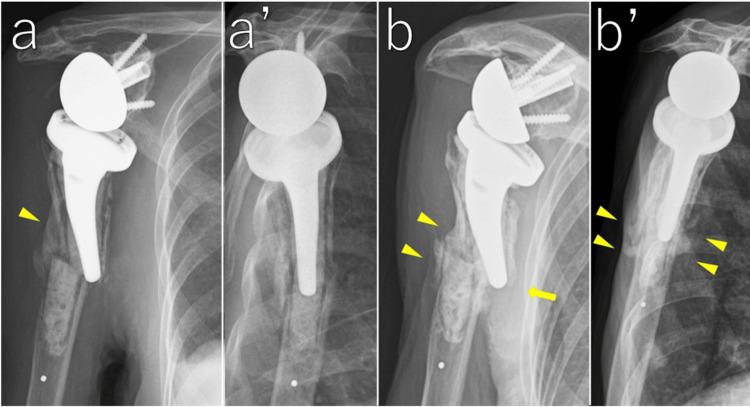
Radiographic course at four weeks and five months after injury Anteroposterior radiograph at four weeks (a) showing callus formation on the lateral cortex (arrowhead). Scapular-Y view at four weeks (a’) showing preserved alignment. Anteroposterior radiograph at five months (b) showing mature callus formation laterally (arrowheads) and poor callus formation medially (arrow). Scapular-Y view at five months (b’) showing good callus formation anteriorly and posteriorly (arrowheads).

No further displacement was observed, and callus formation appeared on radiographs. Pendulum exercises were started (Figure 5). We decided the treatment protocol as follows: weeks 4-6, pendulum exercises; weeks 6-10, active-assisted range of motion; after week 10, active motion as tolerated, with no lifting >1 kg for three months.

Two months after the injury, she was hospitalized at another hospital owing to the exacerbation of heart failure, and follow-up was temporarily interrupted.

Five months after injury

After recovery from heart failure, she returned to our clinic. Radiographs demonstrated maturation of the callus, and fracture site pain had resolved (Figure 5).

Final follow-up (one year after injury)

**Figure 6:**
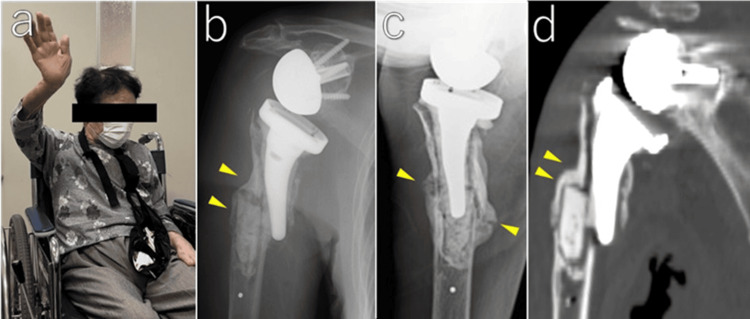
Final follow-up at one year after injury The patient achieved painless active forward elevation (a). Anteroposterior radiograph (b) showing bridging callus formation laterally (arrowheads). Scapular-Y view (c) showing bridging anterior and posterior callus (arrowheads). Coronal CT image of the humerus (d) showing lateral bridging callus extending beyond the cement mantle (arrowheads).

One year after injury, the patient reported no pain, and forward flexion was 100°. Plain radiographs and CT images showed that the distal fragment remained in 20° of valgus, with the stem tip located medial to the distal fragment. Bridging callus formation was observed on the lateral, anterior, and posterior cortex, confirming bone union (Figure 6). She was able to transfer from a wheelchair and dress herself without pain and functional limitation. The patient and her family were satisfied with the outcome.

## Discussion

This case demonstrates successful bone union with conservative treatment in a displaced Wright and Cofield classification type B fracture. Conservative management may therefore be a viable option in selected patients, particularly elderly individuals with multiple comorbidities or high surgical risk.

The acceptable degree of displacement for bone union in type B fractures remains unclear [3]. In type B fractures, the intramedullary stem and cement occupy the medullary canal. This compromises the local blood supply and impairs fracture healing [2]. Consequently, most displaced type B fractures have been treated surgically [6-8], and reports of conservative treatment remain limited. Tansey et al. reported that six of 14 type B fractures (43%) achieved bone union with conservative treatment [1]; however, the bone union rate with conservative treatment remains unclear.

In the present case, the fracture showed angular deformity with an intact lateral cortical hinge, and cortical integrity was preserved on the lateral, anterior, and posterior aspects. This suggests that the periosteum and its blood supply were preserved in these regions. When intramedullary blood flow is reduced by reaming or stem insertion, periosteal blood supply may compensate at the fracture site [9]. In this patient, bone union was achieved at the three cortices with preserved periosteum. In contrast, no callus formation was observed on the disrupted medial cortex. Based on these findings, we hypothesize that preservation of the periosteal soft tissue envelope likely provided adequate compensatory vascularity for achieving bone union with conservative treatment in type B fractures. Therefore, careful assessment of cortical integrity on at least two orthogonal radiographic views is essential when deciding the treatment strategy.

In terms of shoulder function, the degree of deformity that can be tolerated remains controversial. In this case, forward flexion decreased from 150° before injury to 100° after union. However, surgical outcomes are not consistently favorable, as postoperative elevation exceeding 90° is not always achieved [10,11]. Because surgical treatment for type B fractures is highly invasive, careful patient selection and consideration of functional demands are essential. Future case-control studies focusing on functional outcomes are needed to clarify the acceptable limits of deformity following conservative treatment.

Objective functional scores (American Shoulder and Elbow Surgeons (ASES) score, Constant-Murley (Constant) score) were not collected because standardized scoring was not part of the routine follow-up protocol at our institution when this case occurred [12,13]. Future studies incorporating validated functional scoring systems will be essential to clarify functional thresholds that may guide treatment selection in displaced type B fractures.

## Conclusions

We report a case of a displaced type B fracture after RSA that achieved bone union with conservative treatment. Despite valgus displacement and medial stem migration, preservation of cortical continuity on the lateral, anterior, and posterior aspects enabled successful bone union. 

When selecting the treatment for type B fractures, both patient factors and cortical continuity on plain radiographs must be carefully evaluated. Accumulation of additional successful conservative cases is required to better define the indications for conservative treatment.
